# Grouping by feature of cross-modal flankers in temporal ventriloquism

**DOI:** 10.1038/s41598-017-06550-z

**Published:** 2017-08-08

**Authors:** Michaela Klimova, Shin’ya Nishida, Warrick Roseboom

**Affiliations:** 1 Sackler Centre for Consciousness Science, School of Informatics & Engineering, University of Sussex, Brighton, UK; 2 NTT Communication Science Laboratories, Kanagawa, Japan

## Abstract

Signals in one sensory modality can influence perception of another, for example the bias of visual timing by audition: temporal ventriloquism. Strong accounts of temporal ventriloquism hold that the sensory representation of visual signal timing changes to that of the nearby sound. Alternatively, underlying sensory representations do not change. Rather, perceptual grouping processes based on spatial, temporal, and *featural* information produce best-estimates of global event properties. In support of this interpretation, when feature-based perceptual grouping conflicts with temporal information-based in scenarios that reveal temporal ventriloquism, the effect is abolished. However, previous demonstrations of this disruption used long-range visual apparent-motion stimuli. We investigated whether similar manipulations of feature grouping could also disrupt the classical temporal ventriloquism demonstration, which occurs over a short temporal range. We estimated the precision of participants’ reports of which of two visual bars occurred first. The bars were accompanied by different cross-modal signals that onset synchronously or asynchronously with each bar. Participants’ performance improved with asynchronous presentation relative to synchronous - temporal ventriloquism - however, unlike the long-range apparent motion paradigm, this was unaffected by different combinations of cross-modal feature, suggesting that featural similarity of cross-modal signals may not modulate cross-modal temporal influences in short time scales.

## Introduction

An important task for the brain in everyday situations is to process multisensory signals. For our sensory system to construct a coherent representation of the environment, it needs to infer which of the many sensory signals it receives at any given time come from the same source, and how those signals should be combined^1–3^. Much of recent research has focused on studying the circumstances under which signals from different modalities are combined. The way in which sequences of sensory signals are combined or segmented into different perceptual groups appears to be influenced by the spatial, temporal, and featural relationships between them. In the spatial domain, the simple example of perceptual grouping is spatial ventriloquism, whereby vision appears to capture the perceived spatial location of an auditory stimulus^4–7^. A similar phenomenon has been reported in the temporal domain - temporal ventriloquism - wherein the timing of auditory events influences the apparent timing of visual events^8–12^. In an early demonstration of temporal ventriloquism^9^, participants were sequentially presented with two lights, above and below fixation, and performed a temporal order judgment (TOJ) reporting which one had lit up first. The lights were paired with brief, spatially uninformative sounds, with the onsets presented synchronously with the lights or at varying degrees of asynchrony, leading or lagging the lights. When the sounds led the onset of the first light and trailed the onset of the second, participants’ performance on the TOJ improved as though the timing of the onset of the lights had been drawn towards the timing of the sounds, and consequently away from one another.

Building on this early demonstration, Freeman and Driver^8^ provided a further compelling case in support of temporal ventriloquism using a visual apparent motion paradigm. Successive presentation of a visual flash to one side and then the other of a scene can invoke the appearance of directional motion when the visual stimulus-onset asynchrony (vSOA) is shorter in one direction (e.g. left-to-right separated by 333 ms) than the other (right-to-left separated by 666 ms). In their demonstration, Freeman and Driver kept the vSOAs constant (500 ms) such that the visual presentation produced an ambiguous direction of apparent motion. Instead, they varied the SOA of auditory signals that accompanied each visual onset (flankers). The cross-modal flankers could lead the left visual onset and lag the right visual onset, or vice versa. When the flanker lagged the left visual onset and led the right visual onset, participants were more likely to report rightward apparent motion (or vice versa). This occurred despite the fact that the vSOAs were identical on all trials and thus always suggested an ambiguous direction of visual apparent motion. On the basis of these results, a strong account of temporal ventriloquism has been proposed wherein temporal ventriloquism is the result of the sounds changing the timing of the flash at a basic sensory level^8, 13, 14^.

Although much of the focus in cross-modal interactions has been on the influences of these primary sensory dimensions of space and time, there is also evidence that the content or features contained within each signal contribute to the overall multisensory interpretation. For example, it has been shown that the strictness with which participants report synchrony (or asynchrony) between temporally-offset audio and visual signals depends on the type of signal presented (e.g. human face and voice or hammer hitting a peg)^15^. More recently, it was demonstrated^16^ that participants’ performance on an audio-visual TOJ improved when male or female faces were combined with male or female voices compared with when the auditory and visual stimuli were not matched (e.g. a male voice and female face). Moreover, the after-effect induced by audio-visual temporal adaptation is constrained to the content(s) of the adapting stimulus (again, male or female faces and voices)^17, 18^.

**Figure 1:**
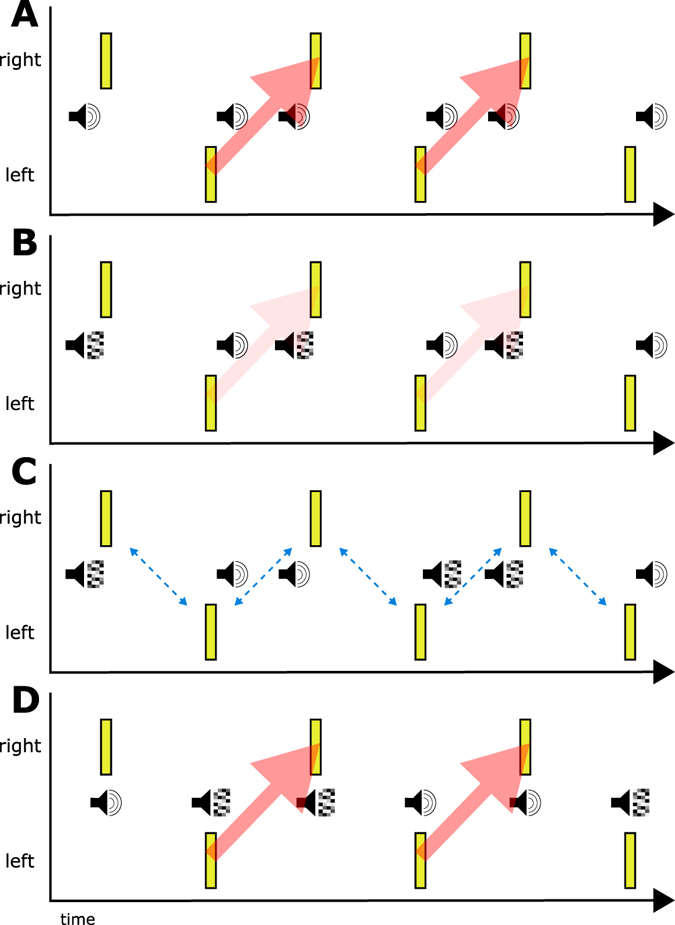
A depiction of the experimental conditions and results in Roseboom *et al*.^19^. (**A**) Identical cross-modal flankers (pure-tone here) presented asynchronously with the visual flashes resulted in a strong impression of motion (indicated by the heavy red arrows). (**B**) When successive flankers differed (audio noise and pure-tone here), the influence of the asynchronous flanker timing was reduced, resulting in a much weaker impression of motion. (**C**) When successive flankers differed by pair and the pairwise features were in conflict with the influence of timing, the perceived motion direction was ambiguous (depicted by the bi-directional blue arrows). (**D**) When successive flankers differed by pairs and there was no temporal difference between visual flashes and cross-modal flankers, this resulted in an impression of rightward motion of a similar magnitude to that apparently driven by temporal ventriloquism. Similar results were obtained when the difference between flanker signals was across-modality (tactile-audio) as when it was within-modality (pure tone-audio noise).

Using a visual apparent motion paradigm similar to that described above, a study by Roseboom *et al*.^19^ investigated the role of cross-modal flanker *feature* on temporal ventriloquism. Participants were presented with visual flashes accompanied by cross-modal flanker signals (either in synchrony or leading/lagging). Successive flankers could be either the same signal (e.g. two audio pure-tone stimuli) or different (e.g. auditory white noise and pure tone). Participants simply had to report the apparent motion direction of the sequence. As in the original demonstration^8^, temporal offset between the flankers and the visual flashes influenced the reported motion direction (Fig. 1A). However, when successive cross-modal flankers differed (e.g. left flash led by audio noise, right flash lagged by pure tone), the influence of flanker *timing* was greatly reduced (Fig. 1B). Moreover, when successive flankers were the same, but successive flanker *pairs* differed (e.g. successive flanker stimulus cycles of noise-noise, pure tone-pure tone; Fig. 1C, and the order of this sequence conflicted with the putative influence of temporal ventriloquism, noise leads-pure tone lags, pure tone leads-noise lags), the ‘temporal ventriloquism’ effect was completely abolished. Finally, in presentations that contained no difference between visual flash and flanker timing (synchronous presentation of cross-modal pairs), and thus remained temporally ambiguous, but the similarity of successive flanker pairings was manipulated (e.g. pure tone-pure tone, noise-noise; Fig. 1D), biases in reported visual apparent motion direction qualitatively similar to those purportedly resulting from ‘temporal ventriloquism’ were found. Similar results were also reported when the flanker signals differed across modality such that, for example, a tactile signal led the left visual flash while an auditory signal lagged the right.

That manipulations of cross-modal flanker signals that changed nothing about timing could severely disrupt and even abolish the apparent temporal ventriloquism, and that similar effects could be produced in the absence of any temporal difference, suggests that a strong interpretation of temporal ventriloquism is unlikely – at least for long range visual apparent motion displays such as used in the two studies described above. Moreover, that similar results were found when the flankers differed but came from the same sensory modality as when they differed across modalities, implies a higher-level, supra-modal influence of perceptual grouping based on feature, rather than a lower-level, within-modality process.

While the results of these experiments clearly demonstrate the importance of perceptual grouping based on featural similarity in determining cross-modal signal combinations, as noted, the experiments in Freeman and Driver^8^ and Roseboom *et al*.^19^ both used long-range apparent motion stimuli (long spatial and temporal intervals). It remains unclear whether a similar influence of grouping by feature would affect temporal ventriloquism occurring on a much shorter time-scale^9, 13^. Indeed, previous research suggests important differences between short and long-range visual apparent motion processing mechanisms^20^. Thus, in this study, we examine whether differences in cross-modal flanker feature similarity can also disrupt temporal ventriloquism on a short temporal scale. To do so, we use a paradigm similar to the classic temporal ventriloquism demonstration provided by Morein-Zamir *et al*.^9^. While the original study always used the same auditory tone for both cross-modal flankers, here we use the same combinations of cross-modal flanker stimuli as used in Roseboom *et al*.^19^, including combinations of audio noise, pure tone audio, and tactile stimuli. By using combinations of flanker stimuli that differ both within (audio noise and pure tone) and across modality (audio and tactile), we can investigate whether, as for the case of long-range visual apparent motion, the role of perceptual grouping within the flanker sequence is similar, regardless of whether the differences are defined by sensory modality or by features within a modality.

**Figure 2:**
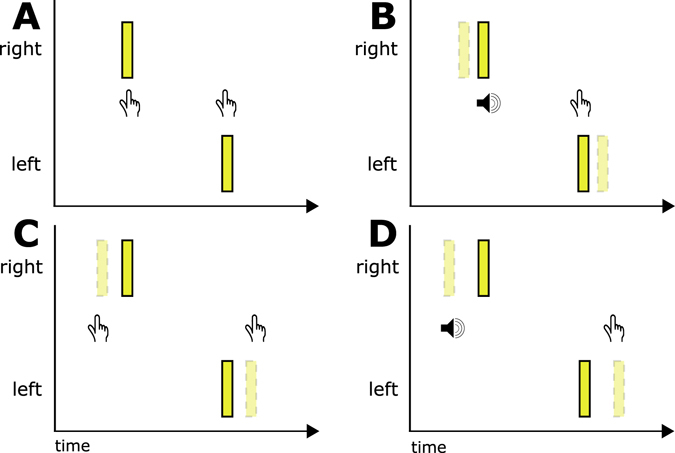
Experimental manipulations of timing and cross-modal flanker similarity in a temporal ventriloquism paradigm based on Morein-Zamir *et al*.^9^. (**A**) Identical flankers presented synchronously with the onset of visual events. (**B**) When cross-modal flankers are presented synchronously but differ in modality (or feature within modality), TOJ performance is consistent with the perceived timing of the visual onsets being further apart. (**C**) When the flankers are identical and presented asynchronously to the visual onsets, TOJ performance is similarly improved (temporal ventriloquism). (**D**) When flankers differ and are presented asynchronously, TOJ performance is improved consistent with the timing of the visual onsets being even further apart.

Consistent with the proposals in Roseboom *et al*.^19^, that both temporal and featural cues contribute to the segmentation of the stimulus sequence, we predict that presentations containing featurally different cross-modal flankers will drive perceptual grouping of events in the temporal ventriloquism display, enhancing perceptual segmentation by comparison with presentations containing identical flankers that rely on temporal information only. Therefore, as depicted in Fig. 2, we expect conditions in which the cross-modal flankers are presented asynchronously with the visual stimuli to demonstrate improved precision - performance consistent with the visual event timing being drawn in the direction of the cross-modal event timing (temporal ventriloquism; Fig. 2C and 2D versus 2A and 2B). We additionally expect that performance will be enhanced in conditions in which the flanker stimuli differ by comparison to when they are the same, regardless of whether a temporal difference is also present (Fig. 2B versus 2A, and 2D versus 2C). Note that although in Fig. 2 we depict the predicted improvement in precision as a shift in timing of the visual flash, consistent with the simple mechanistic suggestion for temporal ventriloquism, this is not what we believe is happening. This depiction is used as a visual convenience to communicate equivalence in performance to a condition with greater temporal separation between visual events, indicating the greater ease with which a TOJ can be made. Finally, based on the results obtained by Roseboom *et al*. when the flanker sequences differed either across modality, or within, we do not expect differences in visual TOJ precision between within and across modality flanker pair conditions.

## Methods

### Participants

20 participants (19 naïve) completed the experiment. All participants reported normal or corrected-to-normal vision and hearing. Naïve participants received ¥1000 per hour for taking part. Informed consent was obtained from all participants, and the research was approved by the ethics committee at Nippon Telegraph and Telephone Corporation. The experiments were carried out in accordance with the guidelines laid out in the Helsinki Declaration.

### Apparatus

The apparatus and basic stimuli were similar to those in Roseboom *et al*.^19^. Visual stimuli were generated with a VSG 2/3 from Cambridge Research Systems (CRS) and presented on a 21 inch Sony Trinitron GDM-F520 monitor, with resolution of 800 × 600 pixels (refresh rate 100 Hz) at a viewing distance of ~105 cm. Auditory and tactile stimuli were generated using a TDT RM1 mobile processor (Tucker-Davis Technologies). Auditory stimuli were presented via a centrally-positioned loudspeaker at ~60 cm distance. Auditory stimulus presentation timing was controlled using a digital line from a VSG Break-out box (CRS) connected to the VSG, which triggered the RM1. Tactile stimuli were delivered via a vibration generator (EMIC Corporation) from ~50 cm distance. Participants rested their right arm on an armrest and their finger was rested on the vibration generator.

### Stimuli

**Figure 3:**
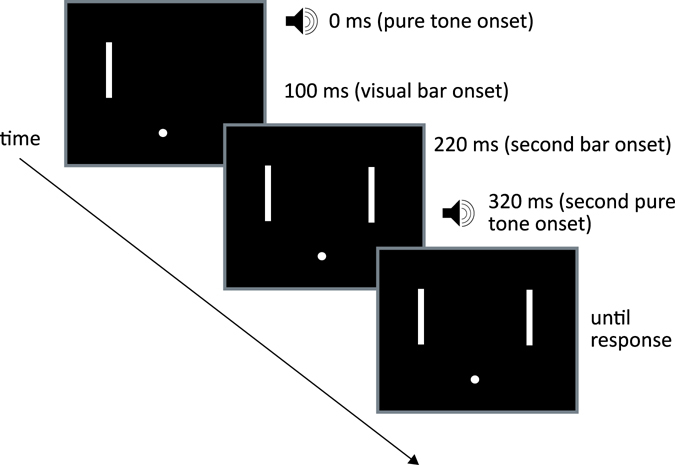
A depiction of a single experimental trial. In this trial, the cross-modal flankers are both pure tone audio, and are asynchronous with respect to the bars, which have a vSOA of 120 ms. In this trial the participant would be expected to report that the left bar appeared first.

The visual stimuli in all conditions were white bars (CIE 1931 x = 0.297, y = 0.321, luminance 123 cd/m^2^; size 0.25 × 1.55 dva). The bars were positioned against a black background with a white central fixation dot, with a distance of 3.35 dva to the left and right of fixation, and 2 dva above fixation. Throughout the experiment, participants were presented with continuous broadband auditory noise from the loudspeaker at ~80 db SPL. Auditory stimuli were composed of a 10 ms pulse, containing 1 ms cosine onset and offset ramps of either a transient amplitude increase in the broadband noise (Noise stimulus, ~85 db SPL) or a 1500 sine-wave carrier (Pure tone stimulus). To mask the noise produced by the tactile stimulator, participants wore Sennheiser HDA200 headphones for passive noise cancelling. Tactile stimuli consisted of a 10 ms pulse, containing 1 ms cosine onset and offset ramps, of a 20 Hz sine-wave carrier. A depiction of a single trial is provided in Fig. 3.

### Task and procedure

The visual inter-stimulus onset asynchrony (vSOA) varied between the following values: −120 ms, −80 ms, −50 ms, −40 ms, −30 ms, −10 ms, then 10 ms up to 120 ms in the same increments. Negative number indicates that the left visual stimulus appeared first. Cross-modal flankers could be presented either synchronously (50% of trials) or asynchronously (50% of trials) with the visual stimuli. When presented asynchronously, they led the onset of the first visual stimulus and lagged the onset of the second visual stimulus by 100 ms. Visual stimuli remained on the screen after their onsets until a response was recorded (see Fig. 3).

There were five types of cross-modal flanker; audio noise only, tactile only, audio noise-tactile, audio pure tone only, and noise-pure tone. Experimental conditions are listed in Table 1. In audio noise only, all cross-modal events were broadband auditory noise. In pure-tone only, all cross-modal events were the pure tone stimuli. In the tactile only, all cross-modal events were tactile stimuli. In the audio-tactile, one visual stimulus was paired with audio noise and the other with a tactile stimulus. In the audio noise-pure tone condition, one visual stimulus was paired with the pure tone stimulus while the other was paired with audio noise. In these combination cross-modal flanker conditions, half of the trials were led by one cross-modal flanker type and followed by the other (e.g. in the audio noise-tactile condition, half the trials had the audio noise signal paired with the first visual stimulus, followed by the tactile signal paired with the second visual stimulus, and the order was reversed for the remaining 50% of trials).

**Table 1:** Cross-modal flanker combinations.

Audio noise only
Audio pure tone only
Tactile
Audio noise – tactile (and reverse)
Audio noise – audio pure tone (and reverse)

In total, participants completed 48 trials for each combination of cross-modal flanker condition, synchrony and vSOA, i.e., 1,152 trials for each cross-modal flanker condition and 5,760 trials in total per participant. Each trial session was comprised of 288 trials (144 trials with synchronous flankers and 144 with asynchronous flankers), with trials completed in a pseudo-random order and taking approximately 10 minutes to complete. Participants completed 20 experimental sessions in a pseudo-random order across two days. In all conditions, the participants’ task was only to report on which side the visual stimulus appeared first, left or right.

### Data availability statement

The data (in.csv format) and R code used for data analysis are available as supplement.

## Results

### Just-noticeable differences

**Figure 4:**
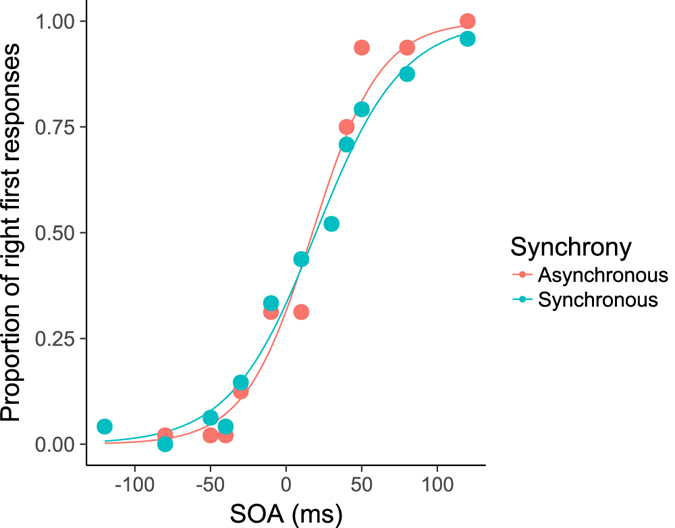
Logistic function fitted to the data from a single representative participant in the audio noise only condition (cross-modal flankers were the same), presented synchronously and asynchronously with the visual onsets. Y-axis represents the proportion of ‘right first’ responses. The steeper slope for reports in the ‘Asynchronous’ presentation condition is consistent with temporal ventriloquism.

We took the proportion of ‘right first’ responses for each participant in each condition and fitted a logistic function using the ‘quickpsy’ package for R^21^. An example of the fitted logistic function to the data from a single participant is shown in Fig. 4 below. From this we calculated the just-noticeable differences (JNDs) and points of subjective equality (PSEs) for each participant, in each condition. JNDs were obtained by taking the half-difference between the vSOA at which 25% of responses were ‘right first’ and the vSOA at which 75% of responses were ‘right first’. The PSEs were estimated as the 50% point of the fitted function and represent the vSOA at which participants were equally likely to respond that either the left or right stimulus appeared first. In addition, for the two combination cross-modal flanker conditions (audio noise-tactile and audio noise-pure tone), the measures were also analysed separately for the two possible directions of pairings (e.g. first flanker was audio-noise, second flanker was tactile, or vice versa). We refer to this data as ‘directional’.

A Bayes factor repeated-measures ANOVA was conducted on the JNDs, with the factors condition (5 levels) and synchrony (synchronous × asynchronous presentation of the cross-modal flanker relative to the visual stimulus). All Bayesian statistical tests were conducted using the ‘BayesFactor’ package for R^22^. Bayes factor calculation represents an alternative to traditional null-hypothesis significance testing by providing an estimate of the relative strength of evidence for two competing hypotheses^23^. The Bayes factor is the ratio of the probability of the observed data under the null hypothesis and the probability of the observed data under the alternative hypothesis^24^. In the Bayes ANOVA, all possible models (main effects, main effects + interactions) are built and compared against the null hypothesis that all effects are 0; the model with the highest Bayes factor value is the favoured model^22^. It is generally accepted that a Bayes factor BF_10_ of 3 or more represents reasonable evidence in support of the alternative hypothesis^25, 26^.

Based on our hypotheses as outlined in the Introduction, we expected a main effect of synchrony such that JNDs will be lower in asynchronous than synchronous conditions, and a synchrony × condition interaction such that JNDs will be smaller in the conditions where cross-modal flankers differ by feature or modality, compared to conditions where they are identical.

The results for the non-directional JND ANOVA showed that the model with two main effects and a condition × synchrony interaction was preferred over either main effects only model (*BF* = 12,802,075 ± 1.78%). An RM-ANOVA was also conducted and showed consistent results; there was a main effect of condition (*F*(4, 171) = 7.19, *p* < 0.001) and synchrony (*F*(1, 171) = 27.72, *p* < 0.001), and a significant synchrony × condition interaction (*F*(4, 171) = 3.78, *p* = 0.006). This was followed up by paired t-tests, FDR-corrected for multiple comparisons. We are only reporting here the t-tests which bear on our hypotheses – the full post-hoc comparisons are available in Supplemental Information which contains raw data and analysis code. Synchronous trials had larger JNDs than asynchronous trials, across all conditions (mean JND = 31.03 ms in synchronous, 26.45 ms in asynchronous), suggesting that performance was improved across all conditions when the cross-modal flanker was asynchronous with respect to the visual stimuli, consistent with the original report^9^.

**Figure 5:**
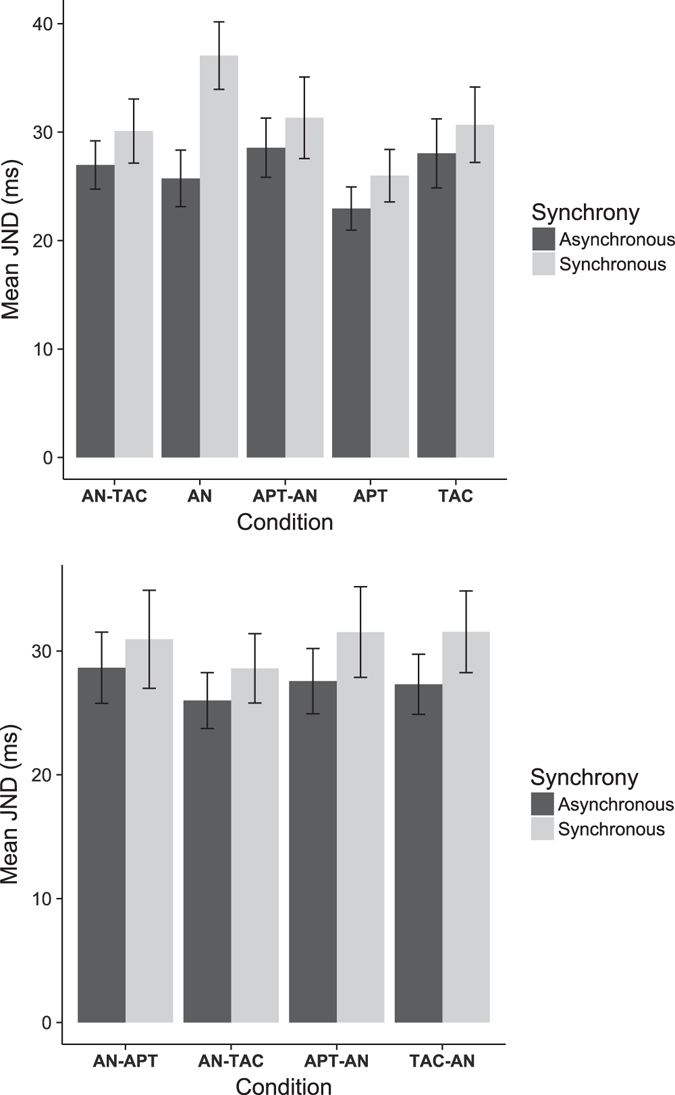
Averaged JND (in milliseconds) for 20 participants for all cross-modal flanker conditions (top panel: AN-TAC = audio noise-tactile condition, AN = audio noise only condition, APT-AN = audio pure tone-audio noise, APT = audio pure tone only, TAC = tactile only) and for the directional conditions (bottom panel: AN-APT = audio noise followed by audio pure-tone, AN-TAC = audio noise followed by tactile, APT-AN = audio pure-tone followed by audio noise, TAC-AN = tactile followed by audio pure tone) based on whether the cross-modal flankers occurred synchronously with the visual onsets, or led the first and lagged the second visual onset by 100 ms. Error bars represent standard error of mean (SEM).

The interaction was shown to be driven by a much larger JND in the audio noise only synchronous condition than in the pure-tone only synchronous (*BF*_*10*_ = 29 ± 0%, *t*(171) = 5.7, *p* < 0.001) and asynchronous (*BF*_*10*_ = 580 ± 0%, *t*(171) = 7.26, *p* < 0.001) conditions, and also between differences in the audio-only and audio-tactile conditions and tactile-only, asynchronous condition. However, JNDs were always smaller in the asynchronous conditions. The results are depicted in Fig. 5 below. Our hypothesis based on the results of Roseboom *et al*.^19^ was that the conditions where the two cross-modal flankers differed in feature/modality would have smaller JNDs than the conditions where cross-modal flankers were identical, for both synchronous and asynchronous presentations. This hypothesis was not supported as the interaction in the ANOVAs was driven by only the difference between one condition with identical flankers (audio noise only) and the rest of the conditions.

Directional JND data (i.e., pure tone-audio noise and audio noise-tactile conditions) were also analysed separately with a Bayes factor 5 (condition) × 2 (synchrony) RM-ANOVA. The model with main effect of synchrony was most strongly supported (mean JND = 27.38 ms in asynchronous, 30.66 ms in synchronous, *BF* = 129.24 ± 0.82%); again in the synchronous trials, JNDs were larger. A traditional RM-ANOVA also showed only the main effect of synchrony as significant (*F*(1, 133) = 15.12, *p* < 0.001). Overall, the JND results point to no difference between the two types of cross-modal flanker (same modality vs. different modalities). This suggests that any differences between the basic signals used in the conditions with different cross-modal flankers did not interact meaningfully with the influence of asynchronous presentation (putatively temporal ventriloquism).

### Bias of visual direction

**Figure 6:**
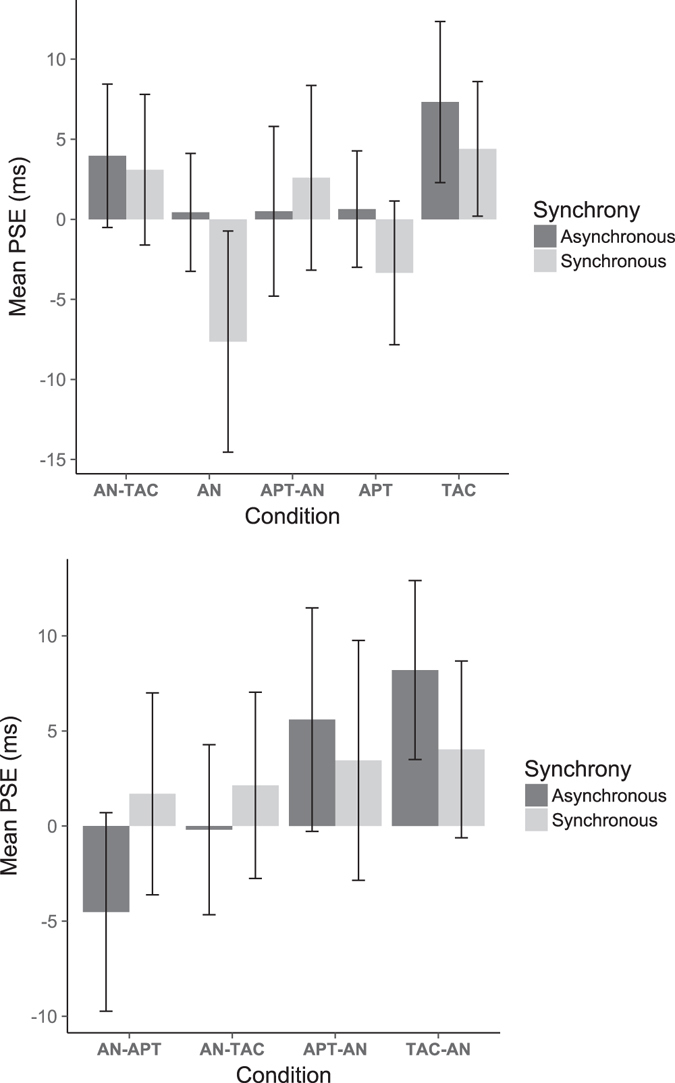
Averaged PSEs across 20 participants, according to the cross-modal flanker condition, for all data (top) and directional data (bottom; see Fig. 5 for condition names) based on whether the cross-modal flankers occurred synchronously with the visual onsets, or led the first and lagged the second visual onset by 100 ms. Error bars are SEM.

Differences in PSE between conditions may indicate differences in the influence of different flanker signals based on perceptual latency, and thus may be masking possible differences in JND above. Consequently, the same statistical analyses were applied to PSEs (see Fig. 6) as for the JNDs above, to test if there was a difference in PSE between when the flanker events were the same and when they differed. A Bayesian RM-ANOVA was conducted on the PSE data. The model with main effects of condition and synchrony, but no interaction, had the most evidence (*BF* = 24,058.16 ± 2.75%). A frequentist RM-ANOVA conducted on all PSE data showed, in agreement with the Bayesian test, a main effect of condition (*F*(4, 171) = 9.37, *p* < 0.001) and a main effect of synchrony (*F*(1,171) = 6.23, *p* = 0.013). PSEs were −0.18 ms in the synchronous conditions, compared to 2.57 ms in the asynchronous conditions, showing a slight bias for participants to report the left visual stimulus as having occurred first when asynchronous flankers were present (*BF*_*10*_ = 5.99 ± 0%). The mean PSEs for the 5 conditions (averaged across synchrony) were as follows: −3.6 ms in audio noise-only, 3.53 ms in audio-tactile, 1.55 ms in pure tone-audio noise, −1.35 ms in pure tone-only, and 5.86 ms in the tactile-only condition.

Identical tests were carried out on the directional PSE data (split by order of cross-modal flankers, as in the directional JNDs). Mean PSEs for the four conditions were: audio-tactile: 0.97 ms, tactile-audio: 6.11 ms, noise-pure tone: −1.4 ms, and pure tone-noise: 4.52 ms. A Bayesian RM-ANOVA showed that the model with a main effect of condition only had the most evidence (*BF* = 240.73 ± 1.87%). Follow-up Bayesian t-tests showed that these differences occurred between the two cross-modal flanker conditions, i.e., there were differences between the noise-pure tone condition and the audio-tactile condition (*BF*_*10*_ = 72.48 ± 0%), between pure tone-noise and tactile-audio (*BF*_*10*_ = 10.88 ± 0%), and between noise-pure tone and tactile-audio (*BF*_*10* = _250.12 ± 0%). There were no systematic differences between the two directions of either of the conditions (e.g. noise-pure tone vs. pure tone-noise). These results suggest that any potential influence of different perceptual latencies for different flanker signals did not affect our interpretation of the role of featural and temporal flanker grouping in this case.

A traditional RM-ANOVA on the directional PSE data showed different results; there was a main effect of condition (*F*(3, 133) = 8.08, *p* = 0.0001) but also a synchrony × condition interaction (*F*(3, 133) = 3.76, *p* = 0.012). Follow-up multiple comparisons (FDR-corrected) showed significant differences between PSEs in audio-tactile asynchronous and tactile-audio asynchronous conditions (*t*(133) = 3.5, *p* = 0.0044), between audio noise-pure tone asynchronous and pure tone-audio noise asynchronous (*t*(133) = 4.22, *p* < 0.001), among other significant differences between conditions and a difference between synchronous and asynchronous PSE in the noise-pure tone condition (*t*(133) = 2.59, *p* = 0.037).

## Discussion and Conclusions

Based on previous results^19^, we expected that presentation of cross-modal flankers that differ by feature would enhance segmentation of the stimulus sequence and improve participants’ performance for determining which visual stimulus occurred first. This enhancement would be revealed by improved precision on visual TOJ for conditions in which cross-modal flankers differed, by comparison with when they were identical and thus provided only temporal cues to segmentation. However, we found that manipulations of flanker feature similarity both between (audition-tactile) and within (audio noise-pure tone) modalities did not affect participants’ ability to discriminate which of two visual stimuli occurred first. Similarly, there was no difference in directional PSEs between the different directions of each pair of cross-modal flankers, indicating that any potential differences in perceptual latency between different signals did not affect the presence of temporal ventriloquism. Only a difference in timing of cross-modal flankers relative to the visual stimuli (synchronous or asynchronous) produced a reliable difference in performance. Therefore, it appears that perceptual grouping based on feature similarity of cross-modal flankers does not influence the perception of visual sequences within the classic temporal ventriloquism paradigm^9^. This would suggest that the additional feature information was not used by our participants in this setup and paradigm. These findings are inconsistent with results previously reported by Roseboom *et al*.^19^ for another case that claimed to demonstrate temporal ventriloquism using long-range visual apparent motion^8^. The results of that study showed that the apparent temporal capture ascribed to temporal ventriloquism was modulated by differences in the flanker feature and thus was likely not consistent with strong accounts of temporal ventriloquism in which the sound changes the timing of the visual stimulus at a sensory level^8, 13, 14^.

In looking for a possible explanation for the difference in results between these experiments, the clearest difference in experimental design is the temporal scale. In the previous study^19^, a long-range visual apparent motion stimulus was used. This stimulus had a single cycle period of approximately 1 second and repeated multiple times. By comparison, in this study, all stimuli were presented within a maximum of 320 ms in a single-shot presentation, with the key visual task occurring in only 120 ms or less. Previous results indicate that temporal ventriloquism-like effects can be found for both short and long temporal scales^13^, though it is also known that long-range and short-range apparent motion, defined by the difference in spatial and temporal extent^20, 27^, differ in the way that they are processed^28–32^. This leaves the possibility that only the higher-level processing involved in long-range visual apparent motion is susceptible to the influence of grouping by cross-modal feature.

However, an explanation based on temporal scale alone is inconsistent with previous results^33^. In a series of experiments examining the cross-modal double-flash illusion (DFI), it was demonstrated that manipulations of feature similarity in the cross-modal flankers interfered with the basic cross-modal effect (a bias of visual number by auditory number). Previous studies had demonstrated that temporal^34^ and spatial proximity^35^ of the multisensory signals were key parameters in determining the strength of the illusion. This was disputed in another study^33^, where the authors reported that when the two flankers differed in either modality (audio-tactile) or feature within modality (audio noise – pure tone), the DFI was abolished, suggesting that – consistent with results from temporal ventriloquism using long-range visual apparent motion - similarity of flankers by feature is important in determining whether the cross-modal signals come from a common source. The DFI occurs within a short temporal interval, with all stimuli being presented within approximately 100 ms. Consequently, it must be possible for featural information to contribute to cross-modal processing of stimuli presented within a short (100 ms) period. This apparent contradiction may be resolvable if estimating number (the task in DFI paradigms) is, in general, a more complex process than determining which of two visual events occurred first. Consistent with this idea, it has been shown that besides being susceptible to the influence of cross-modal feature information, the DFI is modulated by attention^36–38^. In general, perception of numerosity has also been shown to operate largely supramodally^39, 40^ and adaptation of numerosity operates on perceived rather than physical number^41^, features generally indicative of higher-level processing. However, it is thought that several distinct processes underlie numerosity, including processes for smaller (like in the DFI) and larger numbers^42, 43^, and so this is by no means a clear conclusion to draw. In any case, clearly a short stimulus presentation period does not necessarily exclude the possible influence of cross-modal featural information on visual perception.

Another aspect that may differ between short-range apparent motion, and the DFI and long-range apparent motion is the degree of ambiguity in the visual display. It is possible that the short-range, sequential visual order judgment we used was relatively easy for our participants and could be resolved based on temporal cues alone. If temporal ventriloquism follows simple Bayesian inference in spatial, temporal, and featural dimensions^1–3, 44–46^, the influence of feature may be minimal because the information coming from spatial and temporal dimensions is sufficiently precise to make the temporal order judgment with negligible contribution from the additional information. By comparison, the long range visual apparent motion stimuli described in the previous studies were deliberately temporally ambiguous, as the cycle periods were physically matched to produce ambiguous apparent motion (e.g. Freeman and Driver^8^ kept their vSOAs identical and only varied auditory signal timing). Considering the DFI, it has been shown^47^ that the primary determinant of the magnitude of the DFI is participants’ initial visual precision – the less ambiguous the number of visual flashes, the less influence of the cross-modal information. Indeed, using the typical visual stimuli to find a DFI, even in the visual stimulus only condition participants often demonstrate a high error rate, incorrectly reporting if there had been one or two flashes^38^. If a Bayesian inference process is determining the perceived temporal order of events utilising temporal, spatial, *and* featural information, the way to demonstrate an influence of cross-modal featural information on the classic temporal ventriloquism paradigm would simply be to make the task harder. Decreasing the precision with which the sequential visual temporal order judgement is made, for example by embedding the visual onsets within visual noise, should increase the influence of cross-modal featural information relative to the basic spatial and temporal contributions, increasing the difference in performance between different flanker combination conditions. If such an experiment still found no evidence of the influence of cross-modal featural information in determining visual processing, this might indicate, somewhat consistently with the conclusions drawn by^13, 14^, that temporal ventriloquism, at least on short time scales, occurs within relatively low-level sensory processing (e.g. area MT) and relies only on the primary, modality redundant sensory dimensions of space and time. Such studies are necessary to resolve this long-standing issue of precisely what information is available at what processing level when combining information from different sensory modalities.

## Electronic supplementary material

Data analysis script

Dataset 1

Dataset 2
